# An Update on Racial and Ethnic Differences in Neuraxial Anesthesia for Cesarean Delivery

**DOI:** 10.7759/cureus.19729

**Published:** 2021-11-18

**Authors:** Brittany N Burton, Cecilia Canales, Austin L Du, Erin I Martin, Maxime Cannesson, Rodney A Gabriel

**Affiliations:** 1 Anesthesiology and Perioperative Medicine, UCLA Health, Los Angeles, USA; 2 Anesthesiology, UC San Diego Health, San Diego, USA

**Keywords:** health outcomes, neuraxial technique, neuraxial, morbidity, disparities

## Abstract

Background

Racial and ethnic differences in the use of neuraxial anesthesia compared with general anesthesia are less studied, particularly in obstetrical anesthesia. Here, we aimed to provide an update on the association between race and ethnicity, and the use of neuraxial anesthesia for cesarean delivery in the United States (US).

Methods

We used the American College of Surgeons National Surgical Quality Improvement Program (ACS NSQIP) Participant Use Data File 2019. We extracted cases that had a primary surgery defined with Current Procedural Terminology (CPT) code for cesarean delivery (59510, 59514, and 59515) and cesarean after attempted vaginal delivery in parturients with a prior history of cesarean (59618, 59620, and 59622). Multivariable logistic regression was used to report the association of race and ethnicity with primary anesthetic technique.

Results

There were 12,876 parturients included in the study. Compared with White parturients, Black (adjusted odds ratio (aOR) = 0.71, 95% confidence interval (CI): 0.57-0.88, p = 0.001) and American Indian or Alaska Native (aOR = 0.22, 95% CI: 0.12-0.40, p < 0.001) parturients had lower odds of receiving neuraxial compared with general anesthesia. There were no significant differences in the odds of neuraxial anesthesia between non-Hispanic and Hispanic cohorts.

Conclusions

While we do observe racial differences in anesthetic technique, Hispanic patients did not have significantly lower odds of neuraxial anesthesia. This study highlights the importance of an update to prior studies, as the current study suggests a lack of disparity between non-Hispanic and Hispanic parturients. While the results here are encouraging, a multidisciplinary approach is necessary to further address racial disparities.

## Introduction

Childbirth is one of the most anticipated moments for any parent-to-be. What many parents in the United States (US) may not know is that race and ethnicity have been shown to be associated with the anesthetic technique and peripartum complications. According to the Centers for Disease Control and Prevention data, in 2019, nearly 32% of all births in the US were by cesarean delivery [1]. Over 73% of all cesarean deliveries were accomplished using neuraxial anesthesia [2]. Although general anesthesia may be warranted, it is considered to have higher rates of morbidity and mortality when compared with neuraxial anesthesia during cesarean deliveries [3].

In 2007, Glance et al. reported racial disparities in the use of neuraxial anesthesia, noting that Black and Hispanic parturients in labor and delivery were less likely to receive epidural analgesia when compared with White parturients [4]. Nearly 14 years later, we aimed to determine whether racial and ethnic disparities in the US continue in obstetric anesthesia care. Our goal was to evaluate the association between race and ethnicity, and perioperative anesthetic practices in the management of labor and delivery among parturients who underwent cesarean delivery. We used the American College of Surgeons National Surgical Quality Improvement Program (ACS NSQIP) Participant Use Data File 2019 to report an update on the association of self-identified race and ethnicity with neuraxial anesthesia and with postpartum adverse events.

## Materials and methods

Data collection 

The American College of Surgeons National Surgical Quality Improvement Program (ACS NSQIP) Participant Use Data File 2019 was used for this retrospective cohort study. ACS NSQIP is a nationally multi-institutional validated, risk-adjusted surgical outcomes program used to improve and advance perioperative healthcare. It contains 273 variables for each case, including demographic variables, preoperative comorbidities, laboratory data, and 30-day postoperative morbidity and mortality outcome measures [5]. The 2019 data includes patients who underwent cesarean delivery in this year. Per the US Department of Health and Human Services, Office for Human Research Protections, publicly available data does not constitute research with human subjects (as defined under 45 CFR 46:102) [6]. As such, institutional review board approval is not required for this research study. This manuscript adheres to the Strengthening the Reporting of Observational Studies in Epidemiology (STROBE) Enhancing the Quality and Transparency of Health Research (EQUATOR) guidelines.

Data background and quality

An institution trained and certified Surgical Clinical Reviewer (SCR) collects data from medical chart abstraction, and the data is submitted to the ACS NSQIP web-based data collection system. ACS NSQIP undergoes a systemic sampling process called the eight-day cycle to ensure cases have an equal chance of being selected from each day of the week, thereby preventing bias in choosing cases for assessment. ACS NSQIP conducts an Inter-Rater Reliability (IRR) Audit of the selected sites. The IRR Audit involves the review of medical charts, selected either randomly or based on prespecified criteria. Hospital exclusion criteria are imposed when the IRR Audit disagreement rate is over 5%.

Study population

The primary objective of this study was to provide an update of the association of race and ethnicity with the primary outcome (neuraxial anesthesia) and the secondary outcomes (30-day postpartum adverse events for parturients who underwent cesarean delivery). ACS NSQIP collects data for adult parturients. Therefore, all included parturients were ≥18 years old. We extracted cases from ACS NSQIP Participant Use File 2019 that had a primary surgery defined with Current Procedural Terminology (CPT) code for primary cesarean delivery (59510, 59514, and 59515) or cesarean after attempted vaginal delivery in parturients with a prior history of cesarean delivery (59618, 59620, and 59622).

Outcome measures

We evaluated the association of race with several outcome measures. ACS NSQIP contains a variable “ANESTHES,” which was used to define the anesthesia type (primary outcome). Primary anesthesia type was defined as either neuraxial (epidural, spinal, or regional (i.e., epidural, spinal, or combined spinal-epidural)) versus general anesthesia following a cesarean. Thirty-day secondary endpoints included the following: 1) hospital readmission, which is defined as readmission to the same or any hospital for any reason within 30 days of cesarean; 2) reoperation, which is defined as return to the operating room for any reason within 30 days of cesarean; 3) surgical site infection, which included parturients with either superficial, deep, or organ space surgical site infection; 4) sepsis; 5) venous thromboembolism, which included deep venous thrombosis and/or pulmonary embolism; 6) intraoperative and/or postoperative red blood cell transfusion; 7) prolonged hospital length of stay, which is defined as hospital length of stay greater than or equal to the third quartile of the cohort (three days); and 8) mortality, which is defined as death within 30 days of cesarean.

Definition of primary exposure variable

Self-identified race was our primary exposure variable. Thus, the six racial cohorts were as follows: White (reference cohort), Black, Asian, Native Hawaiian or Pacific Islander, American Indian or Alaska Native, and not reported. Self-identified ethnicity was categorized as follows: non-Hispanic (reference cohort), Hispanic, and not reported.

Preoperative variables

We included several potential confounding variables in our analysis. These variables were included given their clinical significance and importance in perioperative health and outcomes. These variables included age, body mass index (BMI), emergency surgery, preoperative sepsis, diabetes, smoking status, congestive heart failure, hypertension, bleeding disorder, American Society of Anesthesiology (ASA) classification, and primary cesarean versus cesarean following vaginal attempt in patients with prior history of cesarean delivery.

Statistical analysis

R (version 4.0.3) was the statistical computing platform used to perform statistical analysis. Analysis of variance (ANOVA) and chi-square tests were used to compare continuous and categorical characteristics, respectively, among all six cohorts. We applied a multivariable logistic regression to the data, in which the dependent variable(s) were the primary and secondary endpoints. The primary independent variables were race and ethnicity. The multivariable analyses were adjusted for emergency cesarean, age, diabetes, active smoker, congestive heart failure, hypertension, diabetes, bleeding disorder, primary cesarean versus cesarean following vaginal attempt in patients with prior history of cesarean delivery, and ASA classification score. The odds ratio (OR) and 95% confidence interval (CI) were presented, and a p < 0.05 was considered statistically significant for outcome measures.

## Results

Parturient characteristics stratified by race and ethnicity

There were 14,029 parturients identified who underwent cesarean from ACS NSQIP Participant Use Data File 2019. After excluding 8% (n = 1153) of the parturients with missing preoperative data, the final sample size was 12,876. Among the study population, Table 1 shows that 10% were Black or African American (n = 1306), 48.7% were White (n = 6279), 6% were Asian (n = 784), 0.67% were Native Hawaiian or Pacific Islander (n = 86), 0.54% were American Indian or Alaska Native (n = 70), and 34% did not report their race (n = 4351). Table 2 shows that 54% were non-Hispanic (n = 6898), 13% were Hispanic (n = 1770), and 33% did not report their ethnicity (n = 4208). For race and ethnicity, there are significant differences in the median age, BMI, emergency (versus nonemergency) cesarean, and prevalence of all preoperative variables, including systemic infection, diabetes, hypertension, bleeding disorder, active smoking, and cesarean after vaginal attempt with prior history of cesarean (p < 0.001).

**Table 1:** Study Characteristics by Race ASA: American Society of Anesthesiology, BMI: body mass index, CHF: congestive heart failure, IQR: interquartile range, SIRS: systemic inflammatory response

	Overall	White	Black	Asian	Native Hawaiian or Pacific Islander	American Indian or Alaska Native	Not Reported	P Value
	12,876	6279	1306	784	86	70	4351	
BMI								<0.001
<20	34 (0.3)	13 (0.2)	2 (0.2)	1 (0.1)	0 (0.0)	0 (0.0)	18 (0.4)	
>20 to <30	4143 (32.2)	1791 (28.5)	294 (22.5)	399 (50.9)	23 (26.7)	16 (22.9)	1620 (37.2)	
>30 to <40	6480 (50.3)	3248 (51.7)	666 (51.0)	353 (45.0)	45 (52.3)	41 (58.6)	2127 (48.9)	
>40 to <50	1835 (14.3)	1029 (16.4)	264 (20.2)	28 (3.6)	11 (12.8)	9 (12.9)	494 (11.4)	
>50 to <60	311 (2.4)	164 (2.6)	55 (4.2)	3 (0.4)	7 (8.1)	2 (2.9)	80 (1.8)	
>60	73 (0.6)	34 (0.5)	25 (1.9)	0 (0.0)	0 (0.0)	2 (2.9)	12 (0.3)	
Emergency Cesarean	3781 (29.4)	1260 (20.1)	295 (22.6)	142 (18.1)	18 (20.9)	19 (27.1)	2047 (47.0)	<0.001
Age (median (IQR))	31 (27–35)	30 (26–34)	29 (25–33)	34 (30–37)	30.5 (27–35)	29 (26–35)	32 (28–35)	<0.001
Systemic Infection								<0.001
No	11913 (92.5)	5801 (92.4)	1235 (94.6)	681 (86.9)	78 (90.7)	62 (88.6)	4056 (93.2)	
SIRS	815 (6.3)	404 (6.4)	62 (4.7)	68 (8.7)	7 (8.1)	7 (10.0)	267 (6.1)	
Sepsis	148 (1.1)	74 (1.2)	9 (0.7)	35 (4.5)	1 (1.2)	1 (1.4)	28 (0.6)	
Diabetes Mellitus								<0.001
No	11876 (92.2)	5848 (93.1)	1205 (92.3)	696 (88.8)	78 (90.7)	61 (87.1)	3988 (91.7)	
Non-Insulin-Dependent	288 (2.2)	131 (2.1)	32 (2.5)	51 (6.5)	5 (5.8)	3 (4.3)	66 (1.5)	
Insulin-Dependent	712 (5.5)	300 (4.8)	69 (5.3)	37 (4.7)	3 (3.5)	6 (8.6)	297 (6.8)	
CHF	8 (0.1)	2 (0.0)	4 (0.3)	0 (0.0)	0 (0.0)	2 (2.9)	0 (0.0)	<0.001
Hypertension	572 (4.4)	239 (3.8)	109 (8.3)	39 (5.0)	4 (4.7)	9 (12.9)	172 (4.0)	<0.001
Bleeding Disorder	133 (1.0)	73 (1.2)	19 (1.5)	7 (0.9)	0 (0.0)	3 (4.3)	31 (0.7)	0.007
Smoker	1238 (9.6)	740 (11.8)	127 (9.7)	12 (1.5)	6 (7.0)	16 (22.9)	337 (7.7)	<0.001
ASA > 3	2278 (17.7)	1117 (17.8)	302 (23.1)	87 (11.1)	15 (17.4)	25 (35.7)	732 (16.8)	<0.001
Cesarean After Vaginal Attempt	277 (2.2)	98 (1.6)	34 (2.6)	27 (3.4)	0 (0.0)	3 (4.3)	115 (2.6)	<0.001

**Table 2:** Study Characteristics by Ethnicity ASA: American Society of Anesthesiology, BMI: body mass index, CHF: congestive heart failure, IQR: interquartile range, SIRS: systemic inflammatory response

	Overall	Non-Hispanic	Hispanic	Not Reported	P Value
n	12,876	6898	1770	4208	
BMI					<0.001
<20	34 (0.3)	10 (0.1)	5 (0.3)	19 (0.5)	
>20 to <30	4143 (32.2)	2141 (31.0)	438 (24.7)	1564 (37.2)	
>30 to <40	6480 (50.3)	3459 (50.1)	982 (55.5)	2039 (48.5)	
>40 to <50	1835 (14.3)	1047 (15.2)	297 (16.8)	491 (11.7)	
>50 to <60	311 (2.4)	191 (2.8)	40 (2.3)	80 (1.9)	
>60	73 (0.6)	50 (0.7)	8 (0.5)	15 (0.4)	
Emergency Cesarean	3781 (29.4)	1424 (20.6)	297 (16.8)	2060 (49.0)	<0.001
Age (median (IQR))	31 (27–35)	30 (26–34)	31 (27–35)	32 (28–35)	<0.001
Systemic Infection					<0.001
No	11913 (92.5)	6369 (92.3)	1604 (90.6)	3940 (93.6)	
SIRS	815 (6.3)	438 (6.3)	130 (7.3)	247 (5.9)	
Sepsis	148 (1.1)	91 (1.3)	36 (2.0)	21 (0.5)	
Diabetes Mellitus					<0.001
No	11876 (92.2)	6380 (92.5)	1621 (91.6)	3875 (92.1)	
Non-Insulin-Dependent	288 (2.2)	182 (2.6)	47 (2.7)	59 (1.4)	
Insulin-Dependent	712 (5.5)	336 (4.9)	102 (5.8)	274 (6.5)	
CHF	8 (0.1)	6 (0.1)	2 (0.1)	0 (0.0)	0.133
Hypertension	572 (4.4)	339 (4.9)	54 (3.1)	179 (4.3)	0.002
Bleeding Disorder	133 (1.0)	88 (1.3)	18 (1.0)	27 (0.6)	0.006
Smoker	1238 (9.6)	844 (12.2)	57 (3.2)	337 (8.0)	<0.001
ASA > 3	2278 (17.7)	1241 (18.0)	336 (19.0)	701 (16.7)	0.063
Cesarean After Vaginal Attempt	277 (2.2)	125 (1.8)	38 (2.1)	114 (2.7)	0.007

Postpartum adverse events after cesarean stratified by race and ethnicity

Among the total sample, 93.3% (n = 12,009) received neuraxial anesthesia for cesarean. Table 3 shows that between races, there was a significant unadjusted difference in the rate of neuraxial anesthesia (p < 0.001), 30-day readmission (p = 0.002), 30-day surgical site infection (p = 0.005), postpartum sepsis (p < 0.001), perioperative transfusion (p < 0.001), and hospital length of stay (p < 0.001). Table 4 shows that across ethnicity, there is a significant unadjusted difference in the rate of neuraxial anesthesia (p = 0.022), surgical site infection (p = 0.001), postpartum sepsis (p < 0.001), perioperative transfusion (p < 0.001), and hospital length of stay (p < 0.001).

**Table 3:** Postpartum Adverse Events After Cesarean Delivery by Race IQR: interquartile range, LOS: length of stay

	Overall	White	Black	Asian	Native Hawaiian or Pacific Islander	American Indian or Alaska Native	Not Reported	P Value
n	12,876	6279	1306	784	86	70	4351	
Neuraxial	12,009 (93.3)	5867 (93.4)	1178 (90.2)	752 (95.9)	78 (90.7)	51 (72.9)	4083 (93.8)	<0.001
Readmission	275 (2.1)	119 (1.9)	47 (3.6)	20 (2.6)	2 (2.3)	3 (4.3)	84 (1.9)	0.002
Reoperation	111 (0.9)	57 (0.9)	12 (0.9)	6 (0.8)	0 (0.0)	4 (5.7)	32 (0.7)	0.772
Surgical Site Infection	474 (3.7)	206 (3.3)	49 (3.8)	18 (2.3)	2 (2.3)	5 (7.1)	194 (4.5)	0.005
Sepsis	154 (1.2)	76 (1.2)	16 (1.2)	43 (5.5)	2 (2.3)	3 (4.3)	14 (0.3)	<0.001
Venous Thromboembolism	16 (0.1)	7 (0.1)	5 (0.4)	0 (0.0)	0 (0.0)	0 (0.0)	4 (0.1)	0.074
Transfusion	362 (2.8)	173 (2.8)	70 (5.4)	32 (4.1)	5 (5.8)	4 (5.7)	78 (1.8)	<0.001
Hospital Stay (median (IQR))	3.05 (2.55)	3 (2–4)	3 (2–4)	3 (2–4)	3 (2–4)	3 (2–4)	2 (2–3)	<0.001
Prolonged LOS	7012 (54.5)	3609 (57.5)	865 (66.2)	534 (68.1)	49 (57.0)	45 (64.3)	1910 (43.9)	<0.001
Mortality	1(0.0)	0 (0.0)		0 (0.0)	0 (0.0)	0 (0.0)	1 (0.0)	0.746

**Table 4:** Postpartum Adverse Events After Cesarean Delivery by Ethnicity IQR: interquartile range, LOS: length of stay

	Overall	Non-Hispanic	Hispanic	Unknown/Not Reported	P Value
n	12,876	6898	1770	4208	
Neuraxial	12,009 (93.3)	6401 (92.8)	1674 (94.6)	3934 (93.5)	0.022
Readmission	275 (2.1)	160 (2.3)	38 (2.1)	77 (1.8)	0.223
Reoperation	111 (0.9)	71 (1.0)	16 (0.9)	24 (0.6)	0.039
Surgical Site Infection	474 (3.7)	218 (3.2)	63 (3.6)	193 (4.6)	0.001
Sepsis	154 (1.2)	110 (1.6)	34 (1.9)	10 (0.2)	<0.001
Venous Thromboembolism	16 (0.1)	8 (0.1)	4 (0.2)	4 (0.1)	0.406
Transfusion	362 (2.8)	230 (3.3)	60 (3.4)	72 (1.7)	<0.001
Hospital Stay (median (IQR))	3.05 (2.55)	3.25 (2.90)	3.17 (2.45)	2.69 (1.87)	<0.001
Prolonged LOS	7012 (54.5)	4238 (61.4)	1002 (56.6)	1772 (42.1)	<0.001
Mortality	1 (0.0)	0 (0.0)	0 (0.0)	1 (0.0)	0.357

Multivariable logistic regression analysis

**Figure 1:**
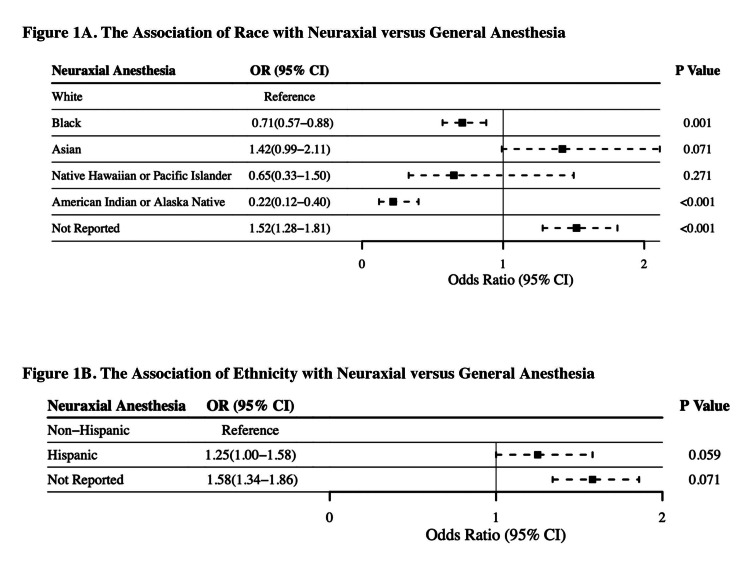
(A,B) Forest Plot of Multivariable Logistic Regression Analysis

Figure 1A shows that compared with White parturients, Black (aOR = 0.71, 95% CI: 0.57-0.88, p = 0.001) and American Indian or Alaska Native (aOR = 0.22, 95% CI: 0.12-0.40, p < 0.001) parturients had lower odds of receiving neuraxial compared with general anesthesia. Figure 1B shows that there was no significant difference in the odds of neuraxial anesthesia between Hispanic and Non-Hispanic cohorts. Table 5 shows the multivariable regression analysis for race and ethnicity with 30-day secondary outcomes.

**Table 5:** Association of Race and Ethnicity with Postpartum Adverse Events LOS: length of stay

	OR (95% CI)	P Value
	30-Day Readmission	
White	Reference	
Black	1.68 (1.17–2.37)	0.003
Asian	1.42 (0.84–2.28)	0.163
Native Hawaiian or Pacific Islander	1.25 (0.2–4.03)	0.761
American Indian or Alaska Native	1.9 (0.46–5.3)	0.288
Not Reported	0.95 (0.7–1.27)	0.71
Non-Hispanic	Reference	
Hispanic	0.96 (0.66–1.36)	0.825
Not Reported	0.73 (0.54–0.97)	0.029
	30-Day Surgical Site Infection	
White	Reference	
Black	1.02 (0.73–1.4)	0.899
Asian	0.87 (0.51–1.39)	0.577
Native Hawaiian or Pacific Islander	0.66 (0.11–2.11)	0.558
American Indian or Alaska Native	2.03 (0.7–4.68)	0.136
Not Reported	1.33 (1.07–1.64)	0.008
Non-Hispanic	Reference	
Hispanic	1.14 (0.85–1.5)	0.384
Not Reported	1.41 (1.14–1.74)	0.001
	30-Day Sepsis	
White	Reference	
Black	1.03 (0.57–1.73)	0.925
Asian	4.49 (2.97–6.72)	<0.001
Native Hawaiian or Pacific Islander	2.11 (0.34–6.89)	0.305
American Indian or Alaska Native	3.2 (0.76–9.06)	0.056
Not Reported	0.22 (0.12–0.39)	<0.001
Non-Hispanic	Reference	
Hispanic	1.21 (0.81–1.77)	0.332
Not Reported	0.12 (0.06–0.22)	<0.001
	Transfusion	
White	Reference	
Black	1.88 (1.4–2.5)	<0.001
Asian	1.49 (0.98–2.19)	0.051
Native Hawaiian or Pacific Islander	2.24 (0.78–5.11)	0.085
American Indian or Alaska Native	1.57 (0.46–3.96)	0.401
Not Reported	0.56 (0.42–0.73)	<0.001
Non-Hispanic	Reference	
Hispanic	1.07 (0.8–1.43)	0.636
Not Reported	0.44 (0.33–0.58)	<0.001
	Prolonged LOS	
White	Reference	
Black	1.34 (1.18–1.52)	<0.001
Asian	1.77 (1.51–2.09)	<0.001
Native Hawaiian or Pacific Islander	0.96 (0.62–1.51)	0.872
American Indian or Alaska Native	1.07 (0.65–1.81)	0.785
Not Reported	0.43 (0.4–0.47)	<0.001
Non-Hispanic	Reference	
Hispanic	0.85 (0.76–0.94)	0.002
Not Reported	0.32 (0.29–0.35)	<0.001

For the subgroup analysis evaluating the association of race and ethnicity with the use of spinal compared with epidural, we found statistically significant differences in anesthetic practice. Compared with White parturients, the odds of the use of epidural (compared with spinal) were significantly higher for Black (aOR = 1.22, 95% CI: 1.05-1.42, p = 0.01061), Asian (aOR = 2.13, 95% CI: 1.78-2.53, p < 0.001), and Native Hawaiian or Pacific Islander (aOR = 2.24, 95% CI: 1.32-3.75, p = 0.002). Compared with Non-Hispanic parturients, the odds of the use of epidural (compared with spinal) were significantly lower for Hispanic parturients (aOR = 0.85, 95% CI: 0.74-0.96, p = 0.011).

## Discussion

In this retrospective study, we show that after adjusting for potential confounders, Black parturients were 29% less to receive neuraxial anesthesia, 68% more likely to have 30-day readmission, 88% more likely to require intraoperative and/or postoperative transfusion, and 34% more likely to have prolonged hospital stay compared with White parturients. American Indian or Alaska Native parturients were 78% less likely to receive neuraxial anesthesia compared with White parturients. Compared with White parturients, Asian parturients had fourfold higher odds of postpartum sepsis, and they had 77% higher odds of prolonged hospital stay. Lastly, there were no differences in surgical site infection among the racial cohorts. There were no differences in anesthetic practice among ethnic cohorts. Compared with non-Hispanic patients, Hispanic patients were 15% less likely to have prolonged hospital stay.

Prenatal comorbidities or pregnancy-related complications may preclude the safe and effective use of neuraxial anesthesia. Early studies show that general anesthesia for cesarean section is associated with postoperative pain and sedation, intraoperative awareness, postpartum hemorrhage, increased risk of fatal aspiration, and failed intubation [3,7]. More recent studies show no significant difference in outcomes for neuraxial versus general anesthesia [8]. In the United Kingdom, the safe and effective transition from epidural analgesia to general anesthesia for cesarean delivery has been recommended as a quality health metric [9].

Quality improvement initiatives, improvements in healthcare, and anesthesia delivered by an obstetrical anesthesiologist led to a dramatic reduction in mortality associated with cesarean delivery by the early 2000s. Hawkins et al. estimated the case fatality rate of general anesthesia during cesarean delivery decreased from 16.8 deaths per million general anesthetics during 1991-1996 to 6.5 deaths per million general anesthetics for 1997-2002 [10]. Potentially avoidable general anesthesia decreased from 6.1% to 3.6% during 2003-2014 among White parturients; however, in non-Hispanic Black parturients, the use of general anesthesia increased from 5.4% to 6.0% during the same time period [10]. Although anesthetic causes now account for less than 2% of all pregnancy-related morbidity and mortality cases in the United States, underrepresented parturients continue to experience worse outcomes.

While one in five Americans is a female who identifies as a minority, racial health disparities are well documented. Tangel et al. conducted an analysis using 2007-2014 data from California, Florida, New York, Maryland, and Kentucky from the Agency for Healthcare Research and Quality (AHRQ) [11]. They showed that after adjusting for socioeconomic status, Black parturients were 44% more likely to receive general anesthesia for cesarean delivery and 45% less likely to receive any analgesia for labor [11]. Glance et al. used the New York State Perinatal registry and showed that compared with White, Black and Hispanic parturients were 22% and 15% less likely to receive epidural analgesia from 1999 to 2003, respectively [4]. Guglielminotti et al. showed that non-Hispanic Black parturients were 27% and Hispanic parturients were 15% more likely to be subjected to general anesthesia without a documented indication when compared with White parturients [3]. Several other studies have shown the Black-White disparity in anesthesia type and perinatal outcomes in obstetrical anesthesiology [12].

While these earlier studies provide a historical perspective, based on the timeframe of data collection and analysis, it is unclear whether these differences in anesthetic practices and health outcomes persist. Because of pervasive racial disparities in healthcare, specifically in obstetrical care, we asked whether race and ethnicity continue to be associated with differences in the primary mode of anesthesia and perinatal outcomes following cesarean delivery. ACS NSQIP is a multi-institutional, well-validated, surgical outcomes registry with data from 719 participating hospitals. This study adds an updated perspective of racial and ethnic disparities in obstetrical anesthesiology, and we provide insight not only into Black-White disparities but also into disparities that exist among other racial cohorts - American Indian or Alaska Native, Asian, and Native Hawaiian or Pacific Islander.

The present findings of increased sepsis and length of stay among Asian parturients warrant additional exploration and possibly clinical caution. In context, these results are consistent with Siddiqui et al.’ observations: compared with White parturients, Asian and Pacific Islander parturients had higher rates of severe morbidities despite higher socioeconomic status and insurance coverage [13]. Notable among these observations is the lower neuraxial anesthesia among Native American or Alaskan Native parturients. Representing more than 25,000 live births per year [14], this group is often included in studies on racial inequities in anesthesia. However, small subpopulation sizes often limit statistically significant conclusions even in nationwide studies [15]. To our knowledge, this represents one of the first nationwide studies identifying disparities in anesthetic technique among Native American and Alaskan Native parturients. Further research into the link between anesthetic technique and perinatal outcomes in this population can inform initiatives to mitigate these disparities.

On November 16, 2020, a statement was released by the American Medical Association to recognize race as a social rather than a biological construct [16]. We agree with this recent argument that race itself may not be a risk factor for poor health outcomes, but rather a proxy of the environment in which people live, cultural traditions, and behavior as it relates to healthcare. Yet, after accounting for socioeconomic factors, healthcare inequities not only persist but also evolve. Other factors that contribute to disparate outcomes include the health system itself, such as the lack of health insurance and geographic barriers to health systems with modern resources. Survey data have also implicated ethnicity as a factor in patient refusal for epidural analgesia [17,18], highlighting the complex interplay between ethnic categorization and cultural health beliefs.

Provider-driven stereotyping and implicit bias contribute to the persistence of health disparities. The practice of medicine with a multidisciplinary approach involving providers, state and federal policymakers, and supportive services (i.e., social workers) will help address health inequity and inequality. Further nationwide studies should explore additional factors associated with peripartum outcomes that may be contributing to the racial trends identified here. For example, future research can explore interactions between race and ethnicity, anesthesia technique, presence of hospital resources, access to prenatal care, and incidence of emergency cesarean section. Elucidating the links between these factors can be especially beneficial for smaller underrepresented populations not typically represented in datasets below the national level.

The strength of ACS NSQIP is it provides primary data from the patients’ medical charts. This is in contrast to many large databases that utilize administrative billing data, which were not originally intended for research purposes. The current study is not without limitations, which is attributed to the retrospective design of the study and the information available in the ACS NSQIP. ACS NSQIP is a voluntary registry with program demands that limit the participation of hospitals. Postpartum complication rates may be different in nonparticipating hospitals, and therefore, the rates may be biased. Factors relevant to obstetrical care are not collected by ACS NSQIP, including, but are not limited to, fetal- and maternal-specific comorbidities, fetal bradycardia, APGAR scores, indication for cesarean, need for crash cesarean, preeclampsia, number of prior pregnancies, prenatal history, severity of comorbidities, intraoperative medications and hemodynamics, sociodemographic data (education, insurance status, median household income, and primary language), and the indication for general anesthesia. Additionally, 34% of the parturients did not report their race, and 33% of the parturients did not report their ethnicity. There are several options for handling missing data, and one commonly used statistical approach involves coding the missing data as “not reported” and including these patients in the analysis [19]. Unfortunately, like many of the approaches to handling missing data, this may lead to biased estimates. Moreover, the relatively small sample size for the Native Hawaiian or Pacific Islander and American Indian or Alaska Native cohorts may decrease the power and increase the likelihood of type II error [20]. Although ACS NSQIP conducts Inter-Rater Reliability (IRR) Audit of selected sites and hospitals are excluded from participating in ACS NSQIP when IRR Audit disagreement rate is over 5%, there is no other data that captures the accuracy of self-reported race or ethnicity, diagnoses, or anesthesia type. As in all retrospective studies, we are not able to report causation but only report associations between variables. The results are not definitive, but rather hypothesis-driving, and future studies are needed to include additional variables that may influence the association between race and primary mode of anesthesia and postpartum adverse events.

## Conclusions

We found a persistence of racial differences in the primary mode of anesthesia and secondary endpoints for cesarean delivery. We did not observe ethnic differences in anesthetic technique or postpartum complications. While racial disparities have been documented throughout the obstetrical anesthesiology literature, within the last decade, there have been several changes to the landscape of healthcare in the United States. We believe that these changes are in part helping to reduce disparities, but further work is needed. We must therefore continue to evaluate and report these issues that affect over 20% of the women population in the United States.
